# The pivotal role of nursing personnel in burn care

**DOI:** 10.4103/0970-0358.70728

**Published:** 2010-09

**Authors:** Elisabeth Greenfield

**Affiliations:** Colonel (Retired), United States Army Nurse Corps,

**Keywords:** Role of nurses, holistic approach, evidence based medicine, critical pathways

## Abstract

The nurses play an important role in the overall management of a burn patient. They must be well versed with the various protocols available that can be used to rationally manage a given situation. The management not only involves medical care but also a psychological assessment of the victim and the family. The process uses a scientific method to combine systems theory with the art of nursing, entailing both problem solving techniques and a decision making process. It involves assessment of the patient to arrive at a diagnosis and then determining the patient goals.An action plan is implemented and is evaluated in the context of patient response. The article discusses many such scenarios in burn patients and outlines the nursing care plans.

Optimal care of the burn patient requires a distinctive multidisciplinary approach. Positive patient outcomes are dependent on the composition of the burn care team and close collaboration among its members. At the centre of this team is the burn nurse, the coordinator of all patient care activities. The complexity and multisystem involvement of the burn patient demand that the burn nurse possess a broad-based knowledge of multisystem organ failure, critical care techniques, diagnostic studies and rehabilitative and psychosocial skills. The nurse oversees the total care of the patient, coordinating activities with other disciplines such as occupational and physical therapy, social services, nutritional services and pharmacy. At the same time, the burn nurse is also a specialist in wound care. As a burn wound heals, either spontaneously or through excision and grafting, the nurse is responsible for wound care and for noting subtle changes that require immediate attention, prevention of infection and pain management. The nurse’s role is continuously expanding. Nurses are conducting nursing research and contributing to evidence-based practice of burn care. Practice guidelines, critical pathways and nursing care plans are all tools that help define and refine the nurse’s role in burn care.

## EVIDENCE-BASED PRACTICE

Recent advances in health care technology, public disclosure and published information as well as a realization that we are obligated to reduce prohibitive health care costs are some of the several factors that have promoted the interest in and development of evidence-based practice or a more objective, scientific approach to health care. Previous standards of care, based largely on experience, are now being used as a control in randomized clinical trials. Both are evaluated using specific endpoints such as cost, benefit and risk.[1] Barnsteiner and Provost[2] suggest that there are both research and nonresearch elements in evidence-based practice. Clinical judgment and critical thinking are equally vital to the process.

## PRACTICE GUIDELINES

Practice guidelines have evolved from the evidence-based practice revolution. They are intended to provide recommendations based on critical reading and interpretation of the current literature for managing specific problems. They attempt to define not only the best but also the most cost-effective treatment. When correctly written, practice guidelines can help minimize practice variances that lead to poor patient outcomes and high health care costs. Because burn centres are few in number and are geographically scattered, there are few burn-focused multicentre trials. Many burn research studies involve only one centre, animal models and small sample sizes. Their limited strength of any demonstrated findings and study conclusions is obvious. There are currently a minimal number of randomized controlled clinical trials that have validated burn clinical care practices. Of the few that do exist, many have been extrapolated from research performed in other critical care patient populations. Recent efforts by the American Burn Association to initiate and support collaboration between burn centres to conduct multicentre trials are on-going. The resulting research studies should generate evidence-based practice and greatly impact future burn care. Additionally, the American Burn Association Committee on Organization and Delivery of Burn Care has published updated Practice Guidelines that were originally published in 2000 as a supplement to the *Journal of Burn Care and Rehabilitation*. The revised and updated recommendations represent the work of the 2004 to 2006 Committee on the Organization and Delivery of Burn Care.[3]

## CRITICAL PATHWAYS

Critical pathways that were developed in the late 1990s as another measure to guide medical and nursing practice are more detailed disease and institution-specific protocols that are usually based on practice guidelines. They define the sequence of standardized, multidisciplinary processes or critical events that must occur in order for a particular patient to move toward desired outcomes within a defined period of time. The goal is to use an interdisciplinary perspective to identify expectations of patient care, improve quality care as demonstrated by improving patient outcomes, decreasing length of stay, decreasing readmissions, decreasing costs and increasing patient satisfaction.[4] They define anticipated length of stay, delineate desired outcomes and goals, provide directions for care, identify the best practice model for a specific group of patients, promote collaboration between disciplines and provide an opportunity for continuous improvement in care delivery.

Critical pathways represent the standard of care in average cases and were developed in response to economic incentives and pressures as they encourage the proper use of resources, which in turn reduces waste of time, energy and material. They promote well-coordinated, well-communicated continuity of care through collaborative practice and facilitate adherence to regulations imposed by regulatory bodies, reduce length of stay and resource utilization and reduce practice variances and adverse outcomes. Table 1 summarizes some of the various purposes that are served by critical pathways.

**Table 1:** Purposes of critical pathways

Improve clinical outcomes
Reduce adverse outcomes
Greater consistency in the delivery of patient care
Improve staff skill levels
Improve basis for performance evaluation
Reduce exposure to liability
Better preparation for accreditation and American Burn Association
Verification
Increase efficiency and productivity

Implementation of critical pathways is challenged by many pros and cons. While they provide a useful guideline in assessment, intervention and evaluation, they must be constantly monitored and updated based on the patient’s response to therapy. Further, they must be individualized for each patient’s needs.[5] They should not to be construed as a cookbook mentality. They are not laws that must be rigidly followed. Contrary to popular belief, they do not annihilate individuality. It is important to remember that they are guidelines that outline the current standards of care. They also provide a useful educational tool for all members of the burn care team as they reflect each team member’s responsibilities. The nurse spends the most time with a patient and is in the best position to monitor progress, report changes and coordinate activities of other team members. Critical pathways are most commonly depicted along two axes, one representing time and one representing aspects of care, including laboratory studies, consult services, nutrition, pharmaceutical support, patient education, etc.

Another useful element of critical pathways is their ability to identify variances, or unexpected events, both positive and negative. The analysis of these variances provides an excellent framework for a quality improvement program and can help focus improvement efforts in any of the four major areas: caregiver or provider, hospital or system, patient or family and/or community variance.

## NURSING DIAGNOSES AND CARE PLANS

During all phases of injury, assessment by the nurse must focus on early detection or prevention of complications associated with moderate to severe burn injury. Frequent monitoring is required to assess indices of essential organ function. A list of the more common actual or potential nursing diagnoses for patients with thermal injuries in the resuscitative, acute and rehabilitative phases of care is presented in Table 2.[6]

**Table 2:** Nursing diagnoses

*Problem statement*	*Etiology*
Ineffective Airway Clearance	Tracheal edema due to inhalation injury
Impaired gas exchange	Interstitial pulmonary edema
Fluid volume deficit	Fluid shifts, dieresis, or evaporative water loss
Altered tissue perfusion	Impaired extremity vascular perfusion with circumferential burns
Risk for infection	Invasive therapy and loss of integument
Hypothermia	Decreased heat production and increased heat loss secondary to thermal injury
Pain	Thermal injury
Ineffective coping	Acute stress from injury and life-threatening crisis
Altered nutrition, less than body requirements	Increased metabolic demands
Impaired skin integrity	Thermal injury
Self-Care deficit	Contractures, therapeutic splinting and positioning
Altered family processes	Potential life style and role changes
Altered body image and self-esteem	Disfigurement or dysfunction following burn injury

The nurse’s goal is to deliver patient-focused care using a holistic approach. In order to accomplish this, the nursing process was introduced in the 1950s and has served as the framework for nursing care delivery ever since. The process uses a scientific method to combine systems theory with the art of nursing. It entails both problem-solving techniques and a decision-making process.[7] The nursing process consists of five steps, which together facilitate the delivery of high-quality, individualized patient care. The five steps are as follows:

Assessment is the first step of the process and is a systemic approach to collecting information about the patient. It includes not only symptoms and physiologic factors but also social, cultural, psychological and spiritual aspects of the patient’s life.

Diagnosis, the second step, is the nurse’s analysis of the assessment. It is sometimes also referred to as needs identification.

Outcomes/planning uses the two previous steps to determine patient goals, both long- and short term, desired outcomes and appropriate nursing interventions. These outcomes and interventions are written as the nursing care plan and serve as a written guide for all health care professionals. An example of a written nursing care plan for the patient in the resuscitative and acute care phases of a major burn injury is provided in by Molter et.al and Ahrns-Klas.[8,9]

Implementation is the action portion of the nursing process and care plan.

Evaluation of both the patient’s response to interventions and progress toward achieving outcome goals is critical. Both need to be documented and the plan of care modified accordingly.

The nursing process is both dynamic and interactive. It is a continuous cycle of logical progression from one step to the next. Because each step relies of the accuracy of the previous step, data must be validated. Clearly, the plan that is developed from the nursing process must be adjusted based on the interactions with other disciplines in order to meet the continuously changing needs of the patient. In creating the care plan, the nurse uses theory, nursing judgment and clinical expertise. In many ways, the nursing process and written plan of care help define the nurse’s role. By using the nursing process, the nurse is able to establish autonomy and a common ground within the practice of nursing through nursing diagnoses. The continuous review of the care plan facilitates evaluation and documentation of outcomes and helps provide the basis for establishing standards of care.

## NURSING DIAGNOSIS 1

Ineffective airway clearance and impaired gas exchange related to tracheal oedema or interstitial oedema secondary to inhalation injury and/or circumferential torso burn manifested by hypoxemia and hypercapnia

### Patient outcomes

- Adequate airway clearance and gas exchange.
- Partial pressure of oxygen >90 mmHg; partial pressure of arterial carbon dioxide <45 mmHg; oxygen saturation >95%.
- Respiration rate 16–20 breaths/min and unlaboured; breath sounds present and clear in all lobes; chest wall excursion symmetrical and adequate.
- Mentation clear; patient mobilises secretions, which are clear to white.

### Nursing interventions

- Monitor oxygen saturation every hour, arterial blood gases as needed; Chest X-ray as ordered
- Assess respiratory rate, character and depth and level of consciousness every hour; breath sounds every 4 h;
  If not intubated, assess for stridor, hoarseness and wheezing every hour
- Administer humidified oxygen as ordered
- Assist patient in coughing and deep breathing every hour while awake;
  Suction every 1–2 h or as needed
  Monitor sputum characteristics and amount
- Turn every 2 h to mobilize secretions
- Elevate head of bed
- Schedule activities to avoid fatigue

### Rationales

- Assess oxygenation and ventilation
- Evaluate respiratory status and Response to treatment
- Expedite elimination of carbon monoxide and prevent/treat hypoxemia
- Promote lung expansion, ventilation, clearing of secretions and clear airway
- Facilitate lung expansion
- Decrease ventillatory effort and dyspnea

## NURSING DIAGNOSIS 2

Adequate fluid volume

Deficient fluid volume secondary to fluid shifts into the interstitium and evaporative loss of fluids from the injured skin

### Patient outcomes

- Heart rate 80–120 beats/min; blood pressure adequate in relation to pulse and urine output; optimal tissue perfusion; nonburn skin warm and pink
- Hourly urine output 30–50 ml/h; 75–100 ml/h in electrical injury; 1 ml/kg/h in children <30 kg body weight
- Weight gain based on volume of fluids given in the first 48 h, followed by diuresis over the next 3–5 days
- Laboratory values within normal limits; urine negative for glucose and ketones

### Nursing interventions

- Monitor: vital signs and urine output q1h until stable; mental status every hour for at least 48 h.
- Titrate fluid requirements to maintain urinary output and haemodynamic stability
- Record daily weight and hourly intake/output measurements; evaluate trends

### Rationales

- Assess perfusion and oxygenation status
- Restore intravascular volume.
- Evaluate fluid loss and replacement.
- Monitor serum electrolytes, glucose, creatinine, haematocrit, blood urea nitrogen as required by patient status
- Evaluate need for fluid and electrolyte replacement resulting from large fluid and protein shifts.

## NURSING DIAGNOSIS 3

Ineffective tissue perfusion related to compression and impaired vascular circulation in the extremities with circumferential burns, as demonstrated by decreased or absent peripheral pulses.

### Patient outcomes

- Adequate tissue perfusion, as manifested by strong peripheral pulses.
- No tissue injury in the extremities secondary to inadequate perfusion from oedema or eschar.

### Nursing interventions

- Assess peripheral pulses every hour for 72 h. Notify the physician of changes in pulses, capillary refill or pain capillary refill or pain
- Elevate upper and lower extremities
- Be prepared to assist with escharotomy or fasciotomy

### Rationales

- Assess peripheral perfusion and the need for escharotomy
- Decrease oedema formation
- Allows oedema expansion and restore peripheral perfusion

## NURSING DIAGNOSIS 4

Acute pain related to burn trauma.

### Patient outcomes

- Relief of pain.
- Identifies factors that contribute to pain. Verbalizes improved comfort level.
- Physiological parameters within normal limits and remain stable after administration of narcotic analgesia.

### Nursing interventions

- Monitor physiological responses to pain, such as increased blood pressure increased heart rate, restlessness and nonverbal cues. Use validated tools in each patient to assess pain and anxiety
- Assess response to analgesics or other interventions
- Evaluate effectiveness of interventions
- Administer analgesic and/or anxiolytic medication as ordered; administer IV during critical care phases
- Medicate patient before bathing, dressing changes and major procedures as needed
- Use nonpharmacological pain-reducing methods as appropriate

### Rationales

- Pain responses are variable and unique to each patient
- Facilitate pain relief. Intramuscular/ intravenous, during critical care phases, medications not consistently absorbed
- Assist patient to perform at higher level as needed of the function
- Reduce need for narcotics

## NURSING DIAGNOSIS 5

Risk for infection related to loss of skin, impaired immune response and invasive therapies.

### Patient outcomes

- Absence of infection.
- No inflamed burn wound margins.
- No evidence of burn wound, donor site or invasive catheter site infection.
- Autograft or allograft skin is adherent to granulation tissue.
- Body temperature and white blood cell count within normal limits.
- Sputum, blood and urine cultures negative.
- Glycosuria, vomiting, ileus,and/or change in mentation absent.

### Nursing interventions

- Assess temperature and vital signs and characteristics of urine and sputum every 1–4 hours
- Monitor white blood cells, burn wound healing status and invasive catheter sites
- Ensure appropriate protective isolation; provide meticulous wound care; educate visitors in burn unit guidelines

### Rationales

- Facilitate early detection of developing infections
- Prevent infection by decreasing exposure, to pathogens

## NURSING DIAGNOSIS 6

### Risk for injury

Gastrointestinal bleeding related to stress response.

### Imbalanced nutrition

Less than body requirements related to paralytic ileus and increased metabolic demands secondary to physiological stress and wound healing.

### Patient outcomes

- Absence of injury and adequate nutrition.
- Decreased gastric motility and ileus resolved.
- No evidence of gastrointestinal haemorrhaging.
- Enteral feedings absorbed and tolerated.
- Daily requirement of nutrients consumed.
- Positive nitrogen balance.
- Progressive wound healing.
- 90% of preburn weight maintained.

### Nursing interventions

- Place nasogastric tube for gastric decompression in >20% TBSA burns
- Assess abdomen and bowel sounds every 8 hours
- Assess NG aspirate (color, quantity, pH, and hemocult blood); monitor stool for hemocult blood
- Administer stress ulcer prophylaxis
- Initiate enteral feeding, and evaluate tolerance.
  Provide high-calorie/protein supplements
  Record all oral intake and count calories
- Schedule interventions and activities to avoid interrupting feeding times
- Monitor weight daily or biweekly

### Rationales

- Prevent nausea, emesis, and aspiration from ileus
- Evaluate resolution of decreased gastric motility
- Facilitate early detection of development of gastrointestinal ulcer
- Prevent stress ulcer development
- Caloric/protein intake must be adequate to maintain positive nitrogen balance and promote healing
- Pain, fatigue, or sedation interferes with desire to eat
- Assess tolerance and response to feeding interventions

## NURSING DIAGNOSIS 7

Risk for hypothermia related to loss of skin and/or external cooling.

### Patient outcome

Normothermia.

Rectal/core temperature 37°C (98.6°F)–38.3°C (101°F).

### Nursing interventions

- Monitor and document rectal/core temperature every 1 to 2 hours; assess for shivering
- Minimize skin exposure; maintain environmental temperatures
- For temperature <37° C (98.6° F), institute rewarming measures

### Rationales

- Evaluate body temperature status
- Prevent evaporative and conductive losses
- Prevent complications

## NURSING DIAGNOSIS 8

Impaired physical mobility and self-care deficit related to burn injury, therapeutic splinting and immobilization requirements after skin graft and/or contractures.

### Patient outcomes

- Physical mobility.
- Demonstrates ability to care for burn wounds.
- No evidence of permanent decreased joint function.
- Verbalises understanding of plan of care.
- Vocation resumed without functional limitations or adjustment to new vocation.

### Nursing interventions

- Perform active and passive range of motion exercises to extremities every 2 hours while awake. Increase activity as tolerated. Reinforce importance of maintaining proper joint movement/function, alignment with splints
- Elevate extremities
- Provide pain relief measures before self-care activities and occupational and physical therapy
- Explain procedures, interventions, and tests in clear, simple, age-appropriate language
- Promote use of adaptive devices as needed to assist in self-care and mobility

### Rationales

- Prevent contractures and loss of
- Decrease edema and promote range of motion and mobility
- Facilitate mobility; assist performance at a higher level of function
- Patient more likely to participate and adhere if understands purpose
- Decrease dependency

## NURSING DIAGNOSIS 9

Risk for ineffective individual coping and disabled family coping related to acute stress of critical injury and potential life-threatening crisis.

### Patient outcomes

- Effective coping.
- Verbalises goals of treatment regimen.
- Demonstrates knowledge of support systems.
- Able to express concerns and fears.
- Patient’s and family’s coping is functional and realistic for the phase of hospitalisation.

### Nursing interventions

- Orient patient and family to unit guidelines and support services; provide written information and reinforce frequently; involve in plan of care. Support adaptive and functional coping mechanisms
- Use interventions to reduce fatigue and pain
- Use social worker for assistance in discharge planning
- Consult psychiatric services for inadequate coping skills or substance abuse treatment
- Promote use of group support sessions

### Rationales

- Decrease fear and anxiety
- Adequate pain control and rest facilitate patient coping
- Provide expert consultation and intervention.
- Assist patient and family in understanding experiences, reactions, and methods of coping

## SUMMARY

The importance of a multidisciplinary approach to patient care cannot be overstated. At the centre of this team is the nurse. The burn nurse’s assessments, observations and evaluations of the patient’s response to interventions are crucial to preventing complications and make the critical difference in patient outcomes.
